# The Proinflammatory Cytokines IL-18, IL-21, and IFN-*γ* Differentially Regulate Liver Inflammation and Anti-Mitochondrial Antibody Level in a Murine Model of Primary Biliary Cholangitis

**DOI:** 10.1155/2022/7111445

**Published:** 2022-03-07

**Authors:** Ya-Fei Xu, Yuan Yao, Min Ma, Shu-Han Yang, Peng Jiang, Jinjun Wang, Koichi Tsuneyama, Chan Wang, Xiangdong Liu, Liang Li, Zhe-Xiong Lian

**Affiliations:** ^1^Chronic Disease Laboratory, School of Medicine, South China University of Technology, Guangzhou 510006, China; ^2^Guangzhou Digestive Disease Center, Guangzhou First People's Hospital, School of Medicine, South China University of Technology, Guangzhou, Guangdong, China; ^3^Key Laboratory of Developmental Genes and Human Diseases, Institute of Life Sciences, Southeast University, Nanjing, Jiangsu, China; ^4^College of Environmental Science and Engineering, Yangzhou University, Yangzhou, Jiangsu, China; ^5^Department of Pathology and Laboratory Medicine, Institute of Biomedical Sciences, Tokushima University Graduate School, Tokushima, Japan; ^6^Guangdong Provincial People's Hospital, Guangdong Academy of Medical Sciences, Guangzhou, China

## Abstract

Primary biliary cholangitis (PBC) is a cholestatic liver disease primarily featured by autoimmune-mediated damage of intrahepatic small- and medium-sized bile ducts. Elevated serum proinflammatory cytokines, serum anti-mitochondrial antibodies (AMAs), liver inflammation, and fibrosis are also hallmarks of PBC disease. However, whether the elevated proinflammatory cytokines play a role in autoimmune cholangitis remains unknown. Herein, we utilized the p40^−/−^IL-2R*α*^−/−^ PBC mouse model to investigate the roles of proinflammatory cytokines IL-18, IL-21, and IFN-*γ* in the onset and progression of PBC. IL-18^−/−^, IFN-*γ*^−/−^, and IL-21^−/−^ mice were crossed with p40^−/−^IL-2Ra^+/-^ mice, respectively, to produce corresponding cytokine-deficient PBC models. Autoantibody level, liver inflammation, and bile duct injury were analyzed. We found that livers from p40^−/−^IL-2R*α*^−/−^ mice exhibit similar transcriptomic characters of PBC patients. In p40^−/−^IL-2R*α*^−/−^ mice, deletion of IL-18 has no remarkable effect on disease progression, while deletion of IL-21 indicates that it is necessary for AMA production but independent of liver inflammation and cholangitis. IFN-*γ* is responsible for both AMA production and liver inflammation in our model. Our results demonstrate that different proinflammatory cytokines can regulate different effector functions in PBC pathogenesis and need to be considered in PBC treatment.

## 1. Introduction

PBC is an autoimmune liver disease, which results from the slow, progressive damage of the small bile ducts [1]. Cumulative bile in the liver leads to apoptosis of hepatocytes, liver inflammation, and activation of hepatic stellate cells. This may lead to scarring, fibrosis, and cirrhosis in the liver and even liver failure in some cases [2]. Elevated serum proinflammatory cytokines are also a feature of PBC [1]. Persistent and excessive proinflammatory cytokine production is a hallmark of many autoimmune diseases and is the target of several therapies [3, 4]. Therefore, understanding the role of cytokines in the occurrence and progression of PBC is of great importance for understanding the pathophysiology of the disease and developing potential therapies.

As liver samples from PBC patients are difficult to obtain, several animal models have been developed for investigating the pathogenesis of PBC, including IL-2R*α*^−/−^ mice, which mimic the early clinical features of human PBC [5]. We reported that deletion of Th1 cytokine IL-12p40 exacerbated PBC-like liver disease with symptoms including increased hepatic portal inflammation, bile duct destruction, and infiltration of liver lymphocytes, which mainly included effector CD4^+^ and CD8^+^ T cells. Particularly, some p40^−/−^IL-2R*α*^−/−^ mice spontaneously developed liver fibrosis, better mimicking the process from inflammation to fibrosis in PBC patients [6]. Therefore, other proinflammatory cytokines may be critical in mediating disease development in p40^−/−^IL-2R*α*^−/−^ mice. According to previous studies of PBC samples and our data on p40^−/−^IL-2R*α*^−/−^ mice, we explored the roles of proinflammatory cytokines: IL-18, IL-21, and IFN-*γ* in the PBC mouse model, respectively.

IL-18 has the ability to promote the development and activation of CD8^+^ effector T cells [7]. It has been reported to promote the development of several autoimmune diseases in mouse models including type 1 diabetes mellitus [8], experimental autoimmune encephalitis [8], and rheumatoid arthritis [9]. IL-18 levels in serum of PBC patients are significantly elevated compared to those in healthy controls or patients with other liver diseases and increased with disease progression [10], suggesting that IL-18 is strongly associated with the progression of PBC.

The expansion, survival, and cytotoxic function of CD8^+^ T cells depend on IL-21 [11, 12]. The combination of IL-21 and IL-21R can regulate the function of a variety of lymphocytes [13] and worsen autoimmune diseases, for example, type I diabetes [14], lupus [15], colitis [16], and rheumatoid arthritis [17] in animal models. Genome-wide association study (GWAS) identified variants in IL21 and IL21R as novel PBC risk loci [18]. This is supported by histochemical studies, which showed that IL-21 and IL-21R were both increased in the liver of PBC patients, positively correlating with the degree of liver inflammation [18]. These studies suggest that IL-21 may play a role in the development of PBC.

The proliferation, differentiation, survival, immune response, apoptosis, and cytolytic function of cytotoxic T lymphocyte (CTL) are reported to be regulated by IFN-*γ* [19–22]. In the livers of PBC patients, IFN-*γ* mRNA-positive cells are significantly increased compared with that of other liver disease patients and healthy controls [23]. Moreover, the expression level of IFN-*γ* mRNA has a strong correlation with an enhanced hepatic portal inflammation, and IFN-*γ* mRNA-positive cells were mainly located around the destructed bile ducts [23]. Besides, IFN-*γ*^+^ cells were stained surrounding the degenerative bile ducts in liver biopsies from PBC patients [24], suggesting that IFN-*γ* is associated with biliary inflammation and injury in PBC.

In our study, we found that deletion of IL-18 in the PBC mouse model did not change disease progression, while deletion of IL-21 restrained the AMA level but not liver inflammation, and deletion of IFN-*γ* restrained both AMA level and liver inflammation.

## 2. Results

### 2.1. Liver Transcriptome of p40^−/−^IL-2R*α*^−/−^ Mice Exhibits Similar Features to Human PBC

**Figure 1:**
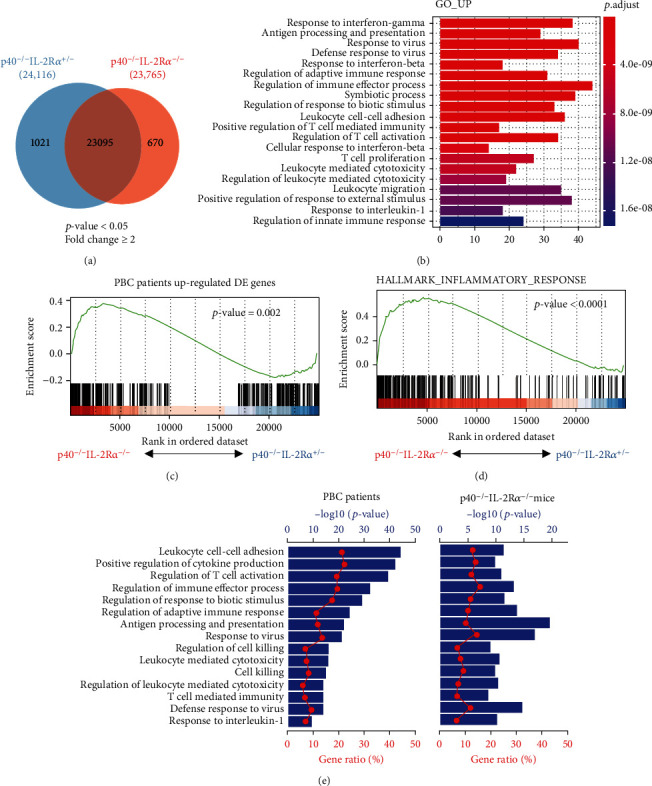
Hepatic gene expression in p40^−/−^IL-2Ra^−/−^ and control mice. (a) Venn diagram of DEGs in livers from p40^−/−^IL-2Ra^−/−^ (*n* = 3) and p40^−/−^IL-2Ra^+/-^ (*n* = 3) mice. (b) GO analysis showing top 20 enriched biological functional pathways of upregulated genes in livers from p40^−/−^IL-2Ra^−/−^ mice. (c, d) GSEA for p40^−/−^IL-2Ra^−/−^ mouse DEGs using gene sets of human PBC DEGs (c) and hallmarks of inflammatory response (d). (e) Canonical enriched pathways of human PBC with p40^−/−^IL-2Ra^−/−^ mice. In one pathway, the ratio of genes captured in this gene set (from PBC patients or p40^−/−^IL-2R*α*^−/−^ mice) to the total number of genes contained in this pathway in the GO database is indicated by “gene ratio” (red line, bottom axis). The “-Log_10_” of the *p* value (blue bar, top axis) was obtained by Fisher's exact test.

We performed RNA sequencing (RNA-seq) of liver transcriptome from p40^−/−^IL-2R*α*^−/−^ and littermate control mice. Livers from p40^−/−^IL-2R*α*^−/−^ mice showed 1691 differentially expressed genes (DEGs) compared with control mice, in which the expressions of 670 genes significantly increased while 1021 genes decreased (Figure 1(a)). The gene ontology (GO) analysis showed that the upregulated DEGs were enriched in genes involved in response to IFN-*γ*, interferon-beta (IFN-*β*), and interleukin-1 (IL-1), positive regulation of T cell-mediated immunity and T cell activation, and leukocyte-mediated cytotoxicity pathways (Figure 1(b)). Consistently, it has been reported that the expressions of IFN-*γ* [23], IFN-*β* [25], and IL-1*β* [25] were upregulated in livers of PBC patients. Therefore, liver transcriptome in p40^−/−^IL-2R*α*^−/−^ mice showed upregulation of proinflammatory cytokines, similar to human PBC.

We then analyzed microarray data of liver biopsies from PBC patients versus normal subjects and identified 386 DEGs. The gene set enrichment analysis (GSEA) revealed that upregulated DEGs in livers of p40^−/−^IL-2R*α*^−/−^ mice were also significantly enriched in the human PBC DEGs (Figure 1(c)). Moreover, the DEGs of p40^−/−^IL-2R*α*^−/−^ mice were significantly enriched in hallmarks of inflammatory response than those of control mice, indicating the liver inflammation feature of this mouse model (Figure 1(d)). Many enriched pathways of human PBC DEGs strongly overlapped with those of p40^−/−^IL-2R*α*^−/−^ mice, including positive regulation of cytokine production, leukocyte-mediated cytotoxicity, and T cell-mediated immunity (Figure 1(e)). These results indicate the similarity between our mouse model and human PBC and highlight that proinflammatory cytokines are involved in PBC development.

### 2.2. Gene Expression Signatures in Livers of p40^−/−^IL-2R*α*^−/−^ Mice Implicate IL-18, IL-21, and IFN-*γ* Pathways

**Figure 2:**
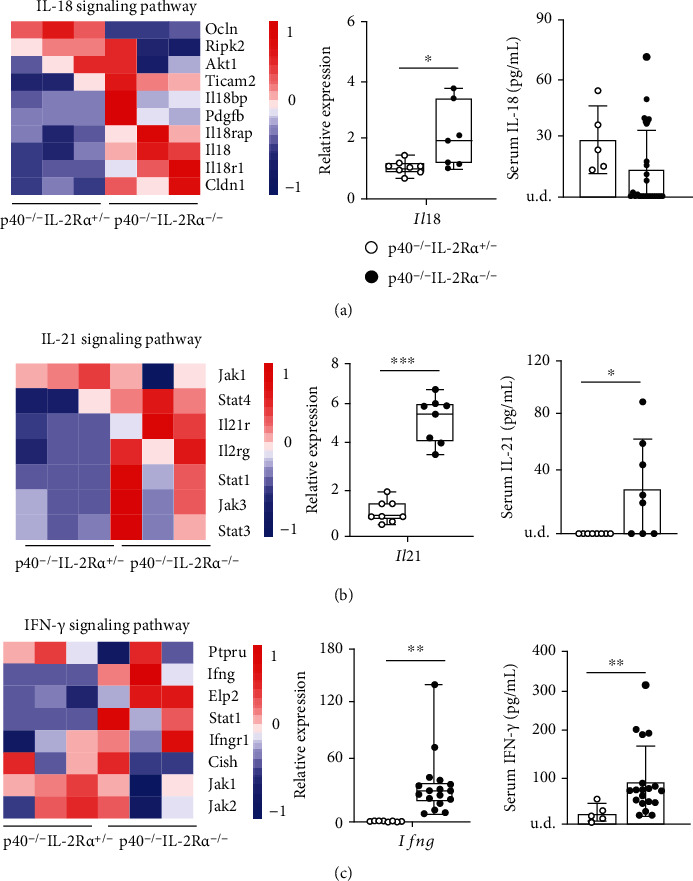
Expression of IL-18, IL-21, and IFN-*γ* was upregulated in p40^−/−^IL-2Ra^−/−^ mice. (a) Heat map showing genes contained in the IL-18 pathway; quantitative real-time PCR analysis for IL-18 mRNA levels in p40^−/−^IL-2Ra^−/−^ (*n* = 7) and p40^−/−^IL-2Ra^+/-^ (*n* = 8) mice and serum IL-18 levels in p40^−/−^IL-2Ra^−/−^ (*n* = 25) and p40^−/−^IL-2Ra^+/-^ (*n* = 5) mice. (b) Heat map showing genes involved in the IL-21 signaling pathway; quantitative real-time PCR analysis for IL-21 mRNA levels in p40^−/−^IL-2Ra^−/−^ (*n* = 8) and p40^−/−^IL-2Ra^+/-^ (*n* = 8) mice and serum IL-21 levels in p40^−/−^IL-2Ra^−/−^ (*n* = 8) and p40^−/−^IL-2Ra^+/-^ (*n* = 8) mice. (c) Heat map showing genes involved in the IFN-*γ* signaling pathway; quantitative real-time PCR analysis for IFN-*γ* mRNA levels in p40^−/−^IL-2Ra^−/−^ (*n* = 17) and p40^−/−^IL-2Ra^+/-^ (*n* = 8) mice and serum IFN-*γ* levels in p40^−/−^IL-2Ra^−/−^ (*n* = 19) and p40^−/−^IL-2Ra^+/-^ (*n* = 5) mice. ^∗^*p* < 0.05,  ^∗∗^*p* < 0.01, and^∗∗∗^*p* < 0.001. Abbreviations: u.d.: undetected.

Furthermore, we explored the gene expression of three proinflammatory cytokine pathways in the livers of p40^−/−^IL-2R*α*^−/−^ mice versus controls. The heat maps showed elevated expression of genes in IL-18, IL-21, and IFN-*γ* signaling pathways in the livers of p40^−/−^IL-2R*α*^−/−^ mice (Figures 2(a)–2(c)). Real-time PCR result confirmed the increased expression of IL-18, IL-21, and IFN-*γ* in the livers of p40^−/−^IL-2R*α*^−/−^ mice (Figures 2(a)–2(c)). In addition, the serum levels of IL-21 and IFN-*γ* in p40^−/−^IL-2R*α*^−/−^ mice significantly increased compared with those of the control while IL-18 may mainly function locally in the liver (Figures 2(a)–2(c)). These findings implicated IL-18, IL-21, and IFN-*γ* in the pathogenesis of livers in the PBC model.

### 2.3. IL-18 Is Not Significantly Involved in Autoimmune Cholangitis in p40^−/−^IL-2R*α*^−/−^ Mice

**Figure 3:**
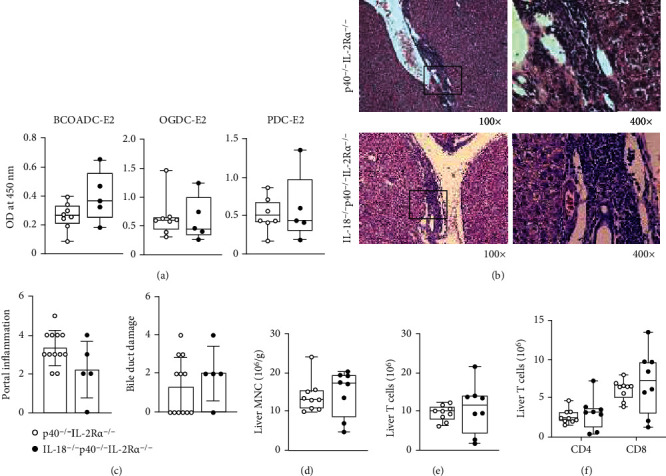
Autoimmune cholangitis in IL-18^−/−^p40^−/−^IL-2Ra^−/−^ mice. (a) Serum AMA levels in IL-18^−/−^p40^−/−^IL-2Ra^−/−^ (*n* = 5) and p40^−/−^IL-2Ra^−/−^ (*n* = 8) mice. (b) Representative H&E of the liver at 100x and 400x magnification. (c) Liver pathological score of IL-18^−/−^p40^−/−^IL-2Ra^−/−^ (*n* = 5) and p40^−/−^IL-2Ra^−/−^ (*n* = 12) mice. (d) Densities of liver MNCs in IL-18^−/−^p40^−/−^IL-2Ra^−/−^ (*n* = 8) and p40^−/−^IL-2Ra^−/−^ (*n* = 9) mice. (e) Numbers of T cells in the liver from IL-18^−/−^p40^−/−^IL-2Ra^−/−^ (*n* = 8) and p40^−/−^IL-2Ra^−/−^ (*n* = 9) mice. (f) Numbers of CD4^+^ and CD8^+^ T cells in the liver from IL-18^−/−^p40^−/−^IL-2Ra^−/−^ (*n* = 8) and p40^−/−^IL-2Ra^−/−^ (*n* = 9) mice. ^∗^*p* < 0.05,  ^∗∗^*p* < 0.01, and^∗∗∗^*p* < 0.001.

We crossed p40^−/−^IL-2R*α*^−/−^ mice with IL-18^−/−^ mice to get IL-18^−/−^p40^−/−^IL-2R*α*^−/−^ mice, for studying the role of IL-18 in PBC-like pathological features. Serologically, the titer of AMA showed no significant difference between IL-18^−/−^p40^−/−^IL-2R*α*^−/−^ mice and p40^−/−^IL-2R*α*^−/−^ mice (Figure 3(a)). Considering the hepatic pathology, knockout of IL-18 did not significantly change the portal inflammation and bile duct damage (Figures 3(b) and 3(c)). Cytologically, the infiltration of liver lymphocytes, cell numbers, and composition of T cells showed no significant change (Figures 3(d)–3(f)). These results suggest that IL-18 does not play a decisive role in liver pathology in this mouse model.

### 2.4. IL-21 Deficiency Decreases the Autoantibody Level but Has No Effect on Liver Inflammation in p40^−/−^IL-2R*α*^−/−^ Mice

**Figure 4:**
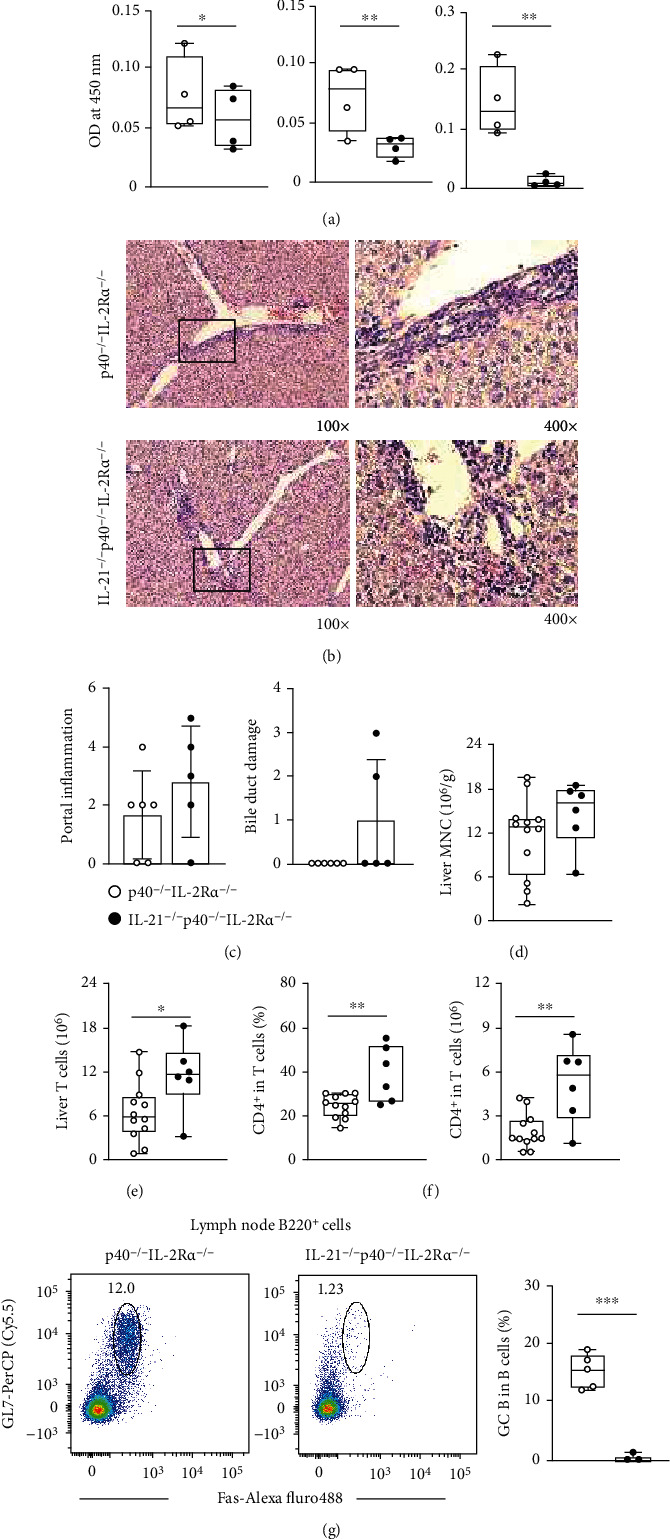
Autoimmune cholangitis in IL-21^−/−^p40^−/−^IL-2Ra^−/−^ mice. (a) Serum AMA levels in IL-21^−/−^p40^−/−^IL-2Ra^−/−^ (*n* = 4) and p40^−/−^IL-2Ra^−/−^ (*n* = 4) mice. (b) Representative H&E of the liver at 100x and 400x magnification. (c) Liver pathological score of IL-21^−/−^p40^−/−^IL-2Ra^−/−^ (*n* = 5) and p40^−/−^IL-2Ra^−/−^ (*n* = 6) mice. (d) Densities of liver MNCs in IL-21^−/−^p40^−/−^IL-2Ra^−/−^ (*n* = 6) and p40^−/−^IL-2Ra^−/−^ (*n* = 12) mice. (e) Numbers of T cells in the liver from IL-21^−/−^p40^−/−^IL-2Ra^−/−^ (*n* = 6) and p40^−/−^IL-2Ra^−/−^ (*n* = 12) mice. (f) Percentages and numbers of CD4^+^ T cells in the liver from IL-21^−/−^p40^−/−^IL-2Ra^−/−^ (*n* = 6) and p40^−/−^IL-2Ra^−/−^ (*n* = 12) mice. (g) Representative flow cytometry plots and percentages of GC B cells in lymph nodes from IL-21^−/−^p40^−/−^IL-2Ra^−/−^ (*n* = 3) and p40^−/−^IL-2Ra^−/−^ (*n* = 5) mice. ^∗^*p* < 0.05,  ^∗∗^*p* < 0.01, and^∗∗∗^*p* < 0.001.

We then crossed p40^−/−^IL-2R*α*^−/−^ mice with IL-21^−/−^ mice to get IL-21^−/−^p40^−/−^IL-2R*α*^−/−^ mice, in order to address the contribution of IL-21 in PBC disease. Comparison of sera from IL-21^−/−^p40^−/−^IL-2R*α*^−/−^ mice and p40^−/−^IL-2R*α*^−/−^ mice revealed that deficiency of IL-21 resulted in significantly downregulated serum AMA levels, suggesting that IL-21 is critical in AMA production in p40^−/−^IL-2R*α*^−/−^ mice (Figure 4(a)). However, liver pathology and immune cell infiltration showed no significant changes (Figures 4(b)–4(d)). Overall, deletion of IL-21 did not affect the liver inflammation in p40^−/−^IL-2R*α*^−/−^ mice. Detection of the mRNA levels of a series of liver fibrosis-related molecules: *Acta2*, *Col1a1*, *Tgfb1*, and *Timp1* in livers from IL-21^−/−^p40^−/−^IL-2Ra^−/−^ mice and controls suggested no significant change of liver fibrosis (Supplementary Figure 3A). T cells showed no significant difference after IL-21 deficiency (Figure 4(e)). Although a significant increase in CD4^+^ T cells was observed in IL-21^−/−^p40^−/−^IL-2R*α*^−/−^ mice (Figure 4(f)), there was no significant change in their activation status as indicated by the percentage of effector memory (Tem) CD4^+^ T cells (Supplementary Figure 1A). Even though the percentage of CD8^+^ T cells in total T cells decreased, the number of CD8^+^ T cells showed no significant change (Supplementary Figure 1B). Therefore, the change of percentages may be attributed to the change of CD4^+^ T cells. Moreover, hepatic CD8^+^ T cell activation showed no significant change as indicated by the percentages of central memory (Tcm) and effector memory (Tem) CD8^+^ T cells (Supplementary Figure 1C). Moreover, the percentages of GC (germinal center) B cells in lymph nodes of IL-21^−/−^p40^−/−^IL-2R*α*^−/−^ mice significantly decreased than that of p40^−/−^IL-2R*α*^−/−^ mice (Figure 4(g)). This result suggests that IL-21 plays a role in the production of PBC autoantibodies, possibly by affecting GC B cells. Overall, IL-21 may be involved in the onset of PBC by participating in the production of autoantibodies, but it has no major role in liver inflammation and bile duct destruction in PBC.

### 2.5. Deficiency of IFN-*γ* Restrains Autoantibody Production and Improves Liver Inflammation in p40^−/−^IL-2R*α*^−/−^ Mice

**Figure 5:**
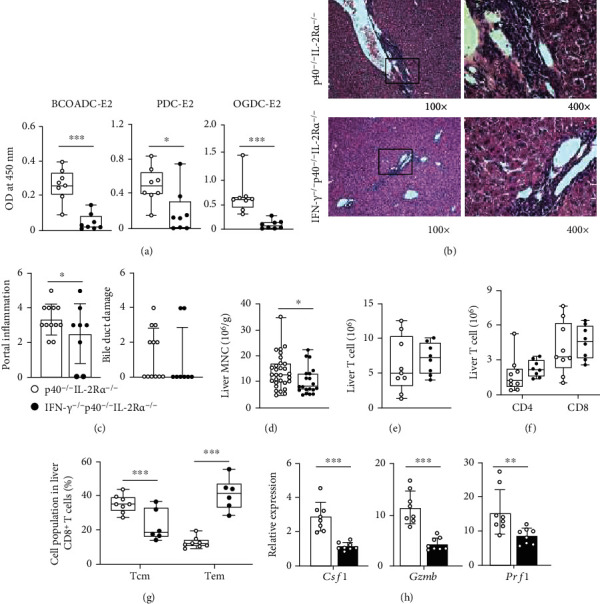
Autoimmune cholangitis in IFN-*γ*^−/−^p40^−/−^IL-2Ra^−/−^ mice. (a) Serum AMA levels in IFN-*γ*^−/−^p40^−/−^IL-2Ra^−/−^ (*n* = 8) and p40^−/−^IL-2Ra^−/−^ (*n* = 8) mice. (b) Representative H&E of the liver at 100x and 400x magnification. (c) Liver pathological score of IFN-*γ*^−/−^p40^−/−^IL-2Ra^−/−^ (*n* = 8) and p40^−/−^IL-2Ra^−/−^ (*n* = 12) mice. (d) Densities of liver MNCs in IFN-*γ*^−/−^p40^−/−^IL-2Ra^−/−^ (*n* = 20) and p40^−/−^IL-2Ra^−/−^ (*n* = 30) mice. (e) Numbers of T cells in the liver from IFN-*γ*^−/−^p40^−/−^IL-2Ra^−/−^ (*n* = 8) and p40^−/−^IL-2Ra^−/−^ (*n* = 9) mice. (f) Numbers of CD4^+^ and CD8^+^ T cells in the liver from IFN-*γ*^−/−^p40^−/−^IL-2Ra^−/−^ (*n* = 8) and p40^−/−^IL-2Ra^−/−^ (*n* = 9) mice. (g) Percentages of CD8^+^ T central memory (Tcm) and effector memory (Tem) cells from IFN-*γ*^−/−^p40^−/−^IL-2Ra^−/−^ (*n* = 6) and p40^−/−^IL-2Ra^−/−^ (*n* = 8) mice. (h) Quantitative real-time PCR analysis for mRNA levels of *Csf1*, *Gzmb*, and *Prf1* in IFN-*γ*^−/−^p40^−/−^IL-2Ra^−/−^ (*n* = 8) and p40^−/−^IL-2Ra^−/−^ (*n* = 8) mice. ^∗^*p* < 0.05,  ^∗∗^*p* < 0.01, and^∗∗∗^*p* < 0.001.

To address the contribution of IFN-*γ* in PBC, we crossed p40^−/−^IL-2R*α*^−/−^ mice with IFN-*γ*^−/−^ mice to produce IFN-*γ*^−/−^p40^−/−^IL-2R*α*^−/−^ mice and measured the PBC disease features in those mice. We found decreased titer of serum AMA in IFN-*γ*^−/−^p40^−/−^IL-2R*α*^−/−^ mice (Figure 5(a)). Histological evaluation of liver tissues from IFN-*γ*^−/−^p40^−/−^IL-2R*α*^−/−^ mice and p40^−/−^IL-2R*α*^−/−^ mice revealed that IFN-*γ* deficiency significantly alleviated the portal inflammation (Figures 5(b) and 5(c)). Quantitative real-time PCR analysis of liver fibrosis-related molecules suggested no significant change of liver fibrosis (Supplementary Figure 3B). Consistently, the cytology data showed a significant reduction of leukocyte infiltration in livers from the IFN-*γ*^−/−^p40^−/−^IL-2R*α*^−/−^ mice than that from p40^−/−^IL-2R*α*^−/−^ littermates (Figure 5(d)). The numbers of liver-infiltrated T, CD4^+^ T, and CD8^+^ T cells remain similar (Figures 5(e) and 5(f)). Although the percentages of central memory (Tcm) CD8^+^ T cells decreased (Figure 5(g)) while those of effector memory (Tem) CD4^+^ T (Supplementary Figure 2A) and CD8^+^ T cells (Figure 5(g)) enhanced, the effector molecules *Csf1*, *Gzmb*, and *Prf1* in livers of IFN-*γ*^−/−^p40^−/−^IL-2R*α*^−/−^ mice expressed significantly lower than those of p40^−/−^IL-2R*α*^−/−^ mice (Figure 5(h)), suggesting that the cytotoxic function of leukocyte is suppressed in the liver. Therefore, IFN-*γ* promotes autoantibody production and liver inflammation in p40^−/−^IL-2R*α*^−/−^ mice.

## 3. Conclusions and Discussion

Deletion of IL-18, IL-21, or IFN-*γ* in p40^−/−^IL-2R*α*^−/−^ mice was supposed to ameliorate autoimmune cholangitis, considering their overexpression in both PBC patients and this mouse model and their reported proinflammatory function. Herein, we showed that deletion of IL-18 changed neither AMA level nor liver inflammation, deletion of IL-21 restrained AMA level but not liver inflammation, and deletion of IFN-*γ* restrained both AMA level and liver inflammation.

90-95% PBC patients have AMAs in serum, and it is disease-specific. However, AMA titer is not associated with PBC disease severity [26]. The contribution of AMA in PBC onset and progression is still unclear even though it has been a key clinical diagnostic indicator of PBC. Based on liver biopsies of PBC patients, CD4^+^ T, CD8^+^ T, and B cells were infiltrated in the inflammatory foci [27, 28]. Their roles during the onset and progression of PBC still remain unknown. Our results suggest that suppression of AMA does not alleviate PBC liver inflammation or bile duct injury when T cell activation is not significantly altered.

We previously reported that depletion of CD8^+^ T cells in p40^−/−^IL-2R*α*^−/−^ mice using anti-CD8a antibody and deletion of CD8a in CD8a^−/−^ mice resulted in remission of salivary gland pathology [29]. Meanwhile, we also observed ameliorated autoimmune cholangitis (data not published), suggesting that CD8^+^ T cells are critical in mediating autoimmune cholangitis in this model. It was reported that IL-18 has the ability to promote CD8^+^ effector T cell development and CTL activity [7]. IL-18 was originally known as an IFN-*γ* inducer [30, 31]. However, in this PBC model, IL-18 neither affected the CD8^+^ T cell-mediated pathogenesis nor the IFN-*γ* production. It was reported that IL-18 induces the IFN-*γ* expression with assistance of IL-12 [32]. The absence of both IL-2R*α* and IL-12p40 in this model may cause IL-18 to lack the necessary synergistic factors to play a proinflammatory role.

IL-21 is well known for its direct effects on B cells and germinal center response [33]. Herein, we reported that deletion of IL-21 significantly suppressed the serum AMA level, which may contribute to the considerable reduction of GC B cells in lymph nodes. It was reported that IL-21 promoted the proliferation, IFN-*γ* expression, and cytolytic function of antigen-specific CD8^+^ T cells and inhibited the responses of non-antigen-specific T cells [34]. Herein, we reported no change in hepatic pathology of IL-21^−/−^p40^−/−^IL-2R*α*^−/−^ mice and no remarkable impact of IL-21 on CD8^+^ T cells. In addition, our previous work suggests that CD8^+^ T cells play a critical role in liver chronic inflammation in p40^−/−^IL-2R*α*^−/−^ mice while neither anti-CD4 antibody therapy nor transgenic CD4 knockout suppresses the onset of the disease (data not published), which might explain why the increase in CD4^+^ T cell infiltration did not cause a significant change of liver inflammation.

In another PBC mouse model, IFN-*γ* ARE-Del^−/−^ mice, the chronic and persistent overexpression of IFN-*γ* led to sex-biased PBC-like autoimmune cholangitis [35]. IFN-*γ* has been reported to be a key factor inducing autoimmunity [36]. It has been hypothesized that IFN-*γ* is upregulated during infection or chemical exposure, leading to a loss of tolerance in genetically susceptible hosts. The proliferation, differentiation, survival, immune response, apoptosis, and cytolytic function of CTL are reported to be related to IFN-*γ* [19–22, 37]. Herein, we found that deletion of IFN-*γ* restrained both the serum AMA level and liver inflammation, probably by repressing the response of CD8^+^ T cells.

In the course of PBC disease, the immune system is in a state of overactivation, and multiple proinflammatory cytokines are upregulated in both patients and model mice. A network of cytokines may drive disease development. Future exploration is needed to resolve which cytokines contribute critically to the onset and development of PBC.

## 4. Materials and Methods

### 4.1. Mice

All mice were raised under specific pathogen-free conditions with an individual ventilation system in the Laboratory Animal Center, South China University of Technology. Mice used in this study were on a C57BL/6J background and sacrificed at 11 to 14 weeks old. Gender was not specifically considered, and both male and female mice were studied. The PBC model p40^−/−^IL-2Ra^−/−^ mice were obtained from p40^−/−^IL-2Ra^+/-^ mice because p40^−/−^IL-2Ra^−/−^ mice are infertile. p40^−/−^IL-2Ra^+/-^ mice were generated from IL-2Ra^−/−^ (B6.129S4-Il2ratm1Dw) mice and p40^−/−^ (B6.129S1-Il12btm1Jm) mice, both initially obtained from The Jackson Laboratory (Bar Harbor, ME). IL-18^−/−^ (JAX: 004130) and IFN-*γ*^−/−^ (JAX: 002287) were initially obtained from The Jackson Laboratory. IL-21^−/−^ (B6/JGpt-Il21^em8Cd6281^/Gpt) mice were obtained from GemPharmatech (Jiangsu, China). These mice were crossed with p40^−/−^IL-2Ra^+/-^ mice, respectively, to get corresponding cytokine-deficient PBC models.

### 4.2. Histology

Mouse liver tissues were fixed with 4% paraformaldehyde, cut into 4 mm slices, and stained with hematoxylin and eosin. The histological scores for portal inflammation or bile duct damage are equal to the sum of the scores of severity and frequency, evaluated by a “blinded” pathologist. Histology quantification was based on the previous reports [38–40].

### 4.3. Flow Cytometry

The isolation of mononuclear cells (MNCs) was performed as described in the previous report [6]. 1 × 10^6^ MNCs were incubated with anti-CD16/CD32 antibody (BioLegend, San Diego, CA) and then stained with antibody cocktails including APC/Cy7-CD45.2 (BioLegend), BV605-CD19 (BioLegend), FITC-CD3 (BioLegend), PE/Cy7-NK1.1 (BioLegend), BUV563-CD4 (BD Biosciences, San Diego, CA), BV711-CD8a (BioLegend), BUV737-CD62L (BD Biosciences), APC-CD44 (BioLegend), PerCP/Cy5.5-GL7 (BioLegend), Alexa Fluor 488-Fas (eBioscience), and BV510-B220 (BioLegend). Flow cytometry was performed with the LSRFortessa flow cytometer (BD Immunocytometry Systems, San Jose, CA), and data were analyzed with FlowJo software (BD Immunocytometry Systems). The full gating strategy is shown in supporting information (Supplementary Figure 4).

### 4.4. Real-Time PCR

Total RNA was extracted from liver tissues using the RNAiso Plus Reagent (TaKaRa, Japan). The PrimeScript RT Reagent Kit (TaKaRa) was used for reverse transcription. Quantitative real-time PCR was performed by LightCycler 96 instrument (Roche, Indianapolis, IN) using Premix Ex Taq (TaKaRa). The PCR primers are listed in Table 1. The relative expression levels of genes except IL-21 were normalized to the housekeeping gene Hprt and calculated by the 2^-*ΔΔ*Ct^ method as previously reported [41]. For IL-21, the PCR product was subjected to SDS agarose gel electrophoresis, and the relative expression level of IL-21 mRNA was calculated based on the gray level of the gel image captured under UV exposure, using ImageJ software (National Institutes of Health, Bethesda, MD, USA).

### 4.5. ELISA

Sera were stored at −80°C until measurement with the Mouse Interleukin 21 ELISA Kit (Cusabio Biotech, Wuhan, China), Mouse IL-18 ELISA Kit (Elabscience, Wuhan, China), or MAX™ Deluxe Set Mouse IFN-*γ* ELISA Kit (BioLegend) according to the manufacturer's instructions. Serum anti-mitochondrial antibodies (AMA) were detected as previously reported [42].

### 4.6. RNA Sequencing and Data Analysis

Total RNA was extracted from liver tissues of p40^−/−^IL-2Ra^−/−^ and p40^−/−^IL-2Ra^+/-^ mice using the RNAiso Plus Reagent (TaKaRa). The cDNA sequencing library was constructed using the Truseq™ RNA sample prep Kit v2 (Illumina, CA, USA) and sequenced using the NovaSeq platform (Illumina). The microarray data set of PBC liver biopsies was downloaded from the National Center for Biotechnology Information/Gene Expression Omnibus (GSE79850). Data were processed through the RPKM matching method and collated as Log_2_ values. Heat maps and GSEA analysis were analyzed in R (Version 4.0) following conventional processes.

### 4.7. Statistical Analysis

For flow cytometry, combined data from 3 or more than 3 independent experiments are shown. For real-time PCR, ELISA, and histology, samples were collected from more than 3 independent experiments and analyzed in one experiment. The number of animals used is stated in figure legends.

We used GraphPad Prism 9 (GraphPad Software, San Diego, CA) for statistical analysis. Symbols represent one individual mouse, and bars are presented as the mean ± standard deviation (SD). The significance of difference was calculated using a two-tailed unpaired Mann-Whitney test unless otherwise stated in figure legends. *p* values were showed as ^∗^*p* < 0.05,  ^∗∗^*p* < 0.01, and^∗∗∗^*p* < 0.001, while *p* values less than 0.05 were not indicated, which were considered not significantly different.

## Figures and Tables

**Table 1:** Real-time PCR primers used in this study.

Genes	Forward (5′-3′)	Reverse (5′-3′)
*Il18*	CAGGCCTGACATCTTCTGCAA	TCTGACATGGCAGCCATTGT
*Il21*	CTTCGTCACCTTATTGACATTGTTG	CCAGGGTTTGATGGCTTGA
*Ifng*	TAGCCAAGACTGTGATTGCGG	AGACATCTCCTCCCATCAGCAG
*Csf1*	ATGAGCAGGAGTATTGCCAAGG	TCCATTCCCAATCATGTGGCTA
*Gzmb*	TGCTCTGATTACCCATCGTCC	GCCAGTCTTTGCAGTCCTTTATT
*Prf1*	TCCCACTCCAAGGTAGCCAA	TGTTAAAGTTGCGGGGGAGG

## Data Availability

The RNA sequencing data have been submitted to the Gene Expression Omnibus (GEO) databases under accession number GSE184066.

## Abbreviations

AMAs: Anti-mitochondrial antibodies
CTL: Cytotoxic T lymphocyte
DEGs: Differentially expressed genes
GC: Germinal center
GEO: Gene Expression Omnibus
GO: Gene ontology
GSEA: Gene set enrichment analysis
GWAS: Genome-wide association study
H&E: Hematoxylin and eosin
MNC: Mononuclear cell
PBC: Primary biliary cholangitis
RNA-seq: RNA sequencing
SD: Standard deviation
Tcm: Central memory T
Tem: Effector memory T
Tfh: Follicular helper T
Treg: Regulatory T.

## Supplementary Materials

Supplementary 1 Supplementary Figure 1: T cell infiltration and activation status in liver from IL-21^−/−^p40^−/−^IL-2Ra^−/−^ mice.

Supplementary 2 Supplementary Figure 2: CD4^+^ T cell activation status in liver from IFN-*γ*^−/−^p40^−/−^IL-2Ra^−/−^ mice.

Supplementary 3 Supplementary Figure 3: liver fibrosis in IL-21^−/−^p40^−/−^IL-2Ra^−/−^ mice and IFN-*γ*^−/−^p40^−/−^IL-2Ra^−/−^ mice.

Supplementary 4 Supplementary Figure 4: flow cytometry gating strategy.
