# Supplementary material for: The Proinflammatory Cytokines IL-18, IL-21, and IFN-γ Differentially Regulate Liver Inflammation and Anti-Mitochondrial Antibody Level in a Murine Model of Primary Biliary Cholangitis
Source: J Immunol Res. 2022 Mar 7;2022:7111445. doi: 10.1155/2022/7111445 (PMC8922149; doi:10.1155/2022/7111445)
Supplement: Supplementary 1 — Supplementary Figure 1: T cell infiltration and activation status in liver from IL-21−/−p40−/−IL-2Ra−/− mice. [file 7111445.f1.pdf]

Supplementary Figure 1

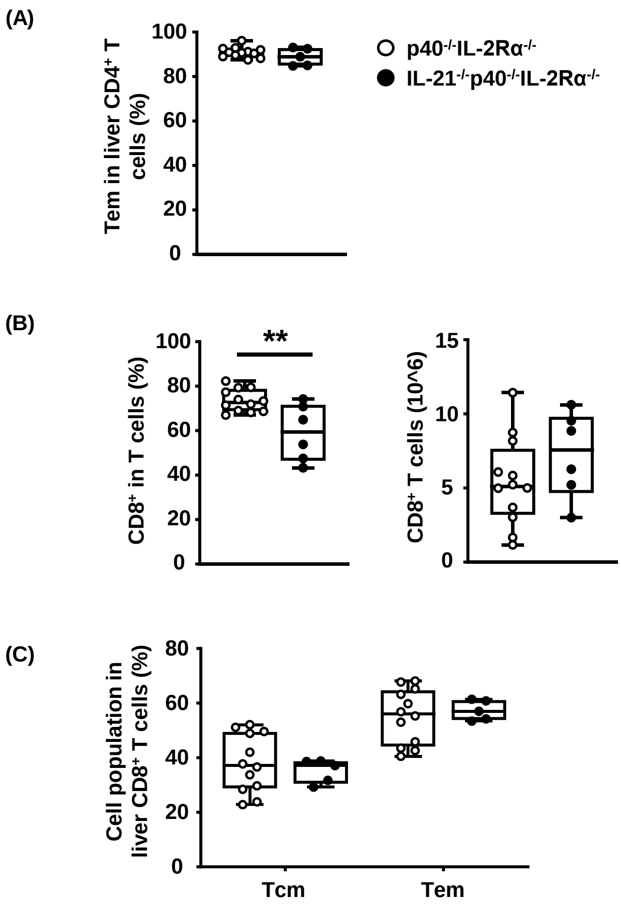

Supplementary Figure 1. T cells infiltration and activation status in liver from IL-21$^{-/-}$p40$^{-/-}$IL-2Ra$^{-/-}$ mice

(A) Percentages of effector memory (Tem) CD4^{+} T cells from IL-21$^{-/-}$p40$^{-/-}$IL-2Ra$^{-/-}$ (n=5) and p40$^{-/-}$IL-2Ra$^{-/-}$ (n=12) mice. **(B)** Percentages and numbers of CD8^{+} T cells in liver from IL-21$^{-/-}$p40$^{-/-}$IL-2Ra$^{-/-}$ (n=6) and p40$^{-/-}$IL-2Ra$^{-/-}$ (n=12) mice. **(C)** Percentages of CD8^{+} T central memory (Tcm) and effector memory (Tem) cells from IL-21$^{-/-}$p40$^{-/-}$IL-2Ra$^{-/-}$ (n=5) and p40$^{-/-}$IL-2Ra$^{-/-}$ (n=12) mice. *$p < 0.05$, **$p < 0.01$, ***$p < 0.001$.
