# Supplementary material for: The Proinflammatory Cytokines IL-18, IL-21, and IFN-γ Differentially Regulate Liver Inflammation and Anti-Mitochondrial Antibody Level in a Murine Model of Primary Biliary Cholangitis
Source: J Immunol Res. 2022 Mar 7;2022:7111445. doi: 10.1155/2022/7111445 (PMC8922149; doi:10.1155/2022/7111445)
Supplement: Supplementary 2 — Supplementary Figure 2: CD4+ T cell activation status in liver from IFN-γ−/−p40−/−IL-2Ra−/− mice. [file 7111445.f2.pdf]

Supplementary Figure 2

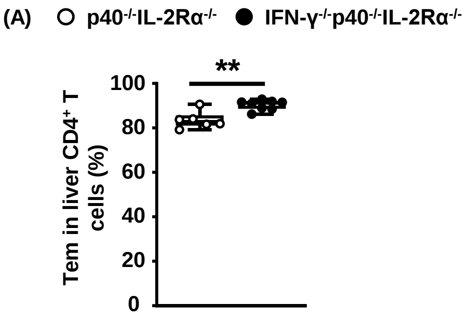

Supplementary Figure 2. CD4$^+$ T cells activation status in liver from IFN-γ$^{-/-}$p40$^{-/-}$IL-2Ra$^{-/-}$ mice

(A) Percentages of effector memory (Tem) CD4$^+$ T cells from IFN-γ$^{-/-}$p40$^{-/-}$IL-2Ra$^{-/-}$ (n=8) and p40$^{-/-}$IL-2Ra$^{-/-}$ (n=6) mice. *p < 0.05, **p < 0.01, ***p < 0.001.
