# Supplementary material for: The Proinflammatory Cytokines IL-18, IL-21, and IFN-γ Differentially Regulate Liver Inflammation and Anti-Mitochondrial Antibody Level in a Murine Model of Primary Biliary Cholangitis
Source: J Immunol Res. 2022 Mar 7;2022:7111445. doi: 10.1155/2022/7111445 (PMC8922149; doi:10.1155/2022/7111445)
Supplement: Supplementary 3 — Supplementary Figure 3: liver fibrosis in IL-21−/−p40−/−IL-2Ra−/− mice and IFN-γ−/−p40−/−IL-2Ra−/− mice. [file 7111445.f3.pdf]

# Supplementary Figure 3

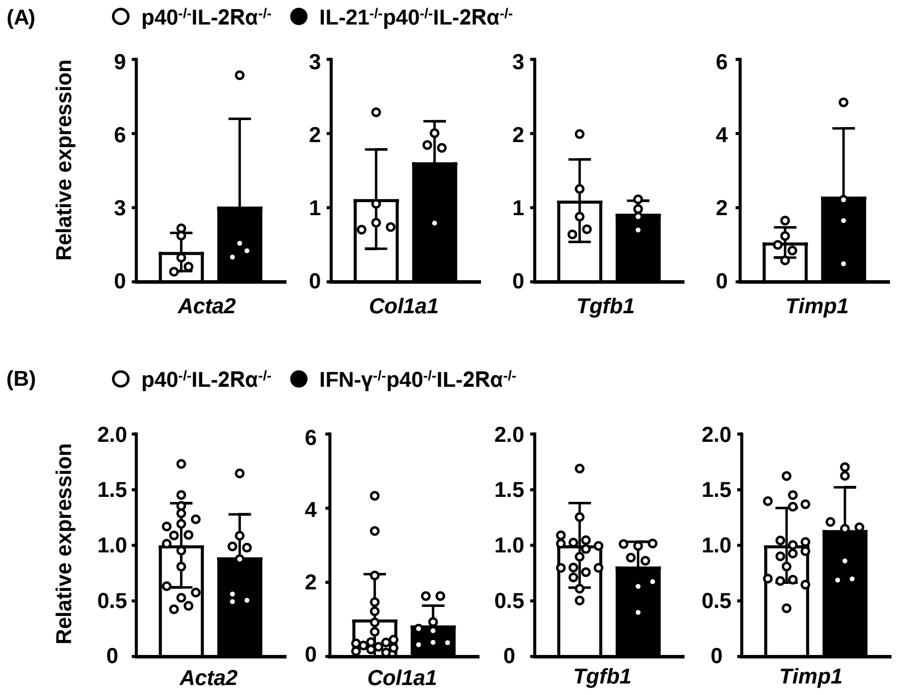


**Supplementary Figure 3. Liver fibrosis in IL-21$^{-/-}$p40$^{-/-}$IL-2Ra$^{-/-}$ mice and IFN-γ$^{-/-}$p40$^{-/-}$IL-2Ra$^{-/-}$ mice**

**(A)** Quantitative real-time PCR analysis of *Acta2*, *Col1a1, Tgfb1* and *Timp1* mRNA levels in livers from IL-21$^{-/-}$p40$^{-/-}$IL-2Ra$^{-/-}$ (n=4) and p40$^{-/-}$IL-2Ra$^{-/-}$ (n=5) mice. **(B)** Quantitative real-time PCR analysis of *Acta2*, *Col1a1, Tgfb1* and *Timp1* mRNA levels in livers from IFN-γ$^{-/-}$p40$^{-/-}$IL-2Ra$^{-/-}$ (n=8) and p40$^{-/-}$IL-2Ra$^{-/-}$ (n=17) mice. *p < 0.05, **p < 0.01, ***p < 0.001.
