# Supplementary material for: The Proinflammatory Cytokines IL-18, IL-21, and IFN-γ Differentially Regulate Liver Inflammation and Anti-Mitochondrial Antibody Level in a Murine Model of Primary Biliary Cholangitis
Source: J Immunol Res. 2022 Mar 7;2022:7111445. doi: 10.1155/2022/7111445 (PMC8922149; doi:10.1155/2022/7111445)
Supplement: Supplementary 4 — Supplementary Figure 4: flow cytometry gating strategy. [file 7111445.f4.pdf]

Supplementary Figure 4

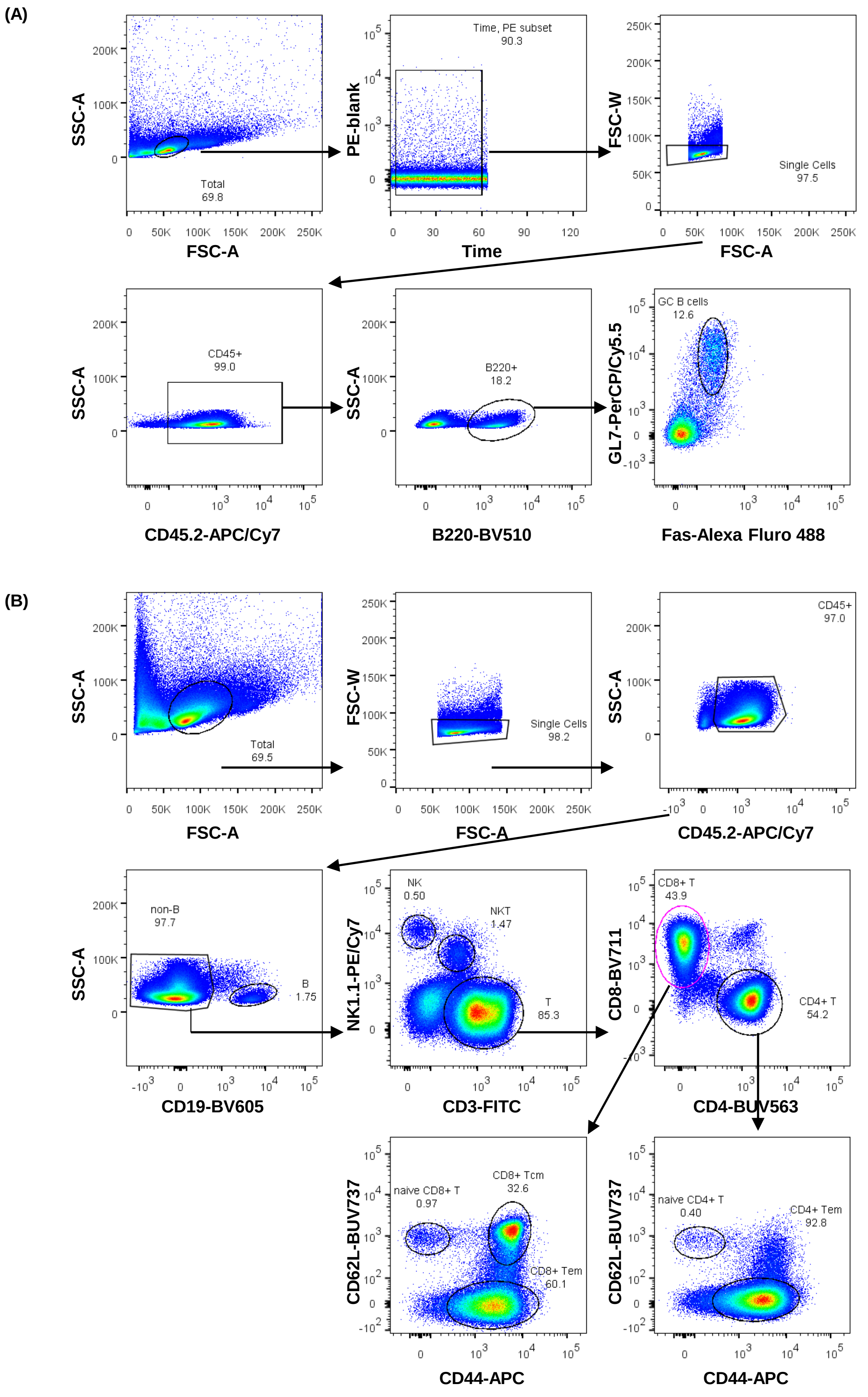

Supplementary Figure 4. Flow cytometry gating strategy
(A) The full gating strategy of T cell subsets. **(B)** The full gating strategy of GC B cells.
